# Impact of dietary lysophospholipids supplementation on growth performance, meat quality, and lipid metabolism in finishing bulls fed diets varying in fatty acid saturation

**DOI:** 10.1186/s40104-024-01138-w

**Published:** 2025-01-09

**Authors:** Meimei Zhang, Haixin Bai, Ruixue Wang, Yufan Zhao, Wenzhu Yang, Jincheng Liu, Yonggen Zhang, Peixin Jiao

**Affiliations:** 1 College of Animal Science and Technology, Northeast Agricultural University, Harbin, 150030 People’s Republic of China; 2 Lethbridge Research and Development Centre, Lethbridge, T1J 4B1 Canada

**Keywords:** Bulls, Fatty acids, Lipid metabolism, Lysophospholipids, Meat quality

## Abstract

**Background:**

The objective of this study was to evaluate the effects of dietary fatty acids (FA) saturation and lysophospholipids supplementation on growth, meat quality, oxidative stability, FA profiles, and lipid metabolism of finishing beef bulls. Thirty-two Angus bulls (initial body weight: 623 ± 22.6 kg; 21 ± 0.5 months of age) were used. The experiment was a completely randomized block design with a 2 × 2 factorial arrangement of treatments: 2 diets with FA of different degree of unsaturation [high saturated FA diet (HSFA) vs. high unsaturated FA diet (HUFA)] combined with (0.075%, dry matter basis) and without lysophospholipids supplementation. The bulls were fed a high-concentrate diet (forage to concentrate, 15:85) for 104 d including a 14-d adaptation period and a 90-d data and sample collection period.

**Results:**

No interactions were observed between dietary FA and lysophospholipids supplementation for growth and meat quality parameters. A greater dietary ratio of unsaturated FA (UFA) to saturated FA (SFA) from 1:2 to 1:1 led to lower DM intake and backfat thickness, but did not affect growth performance and other carcass traits. Compared with HSFA, bulls fed HUFA had greater shear force in *Longissimus thoracis* (LT) muscle, but had lower intramuscular fat (IMF) content and SOD content in LT muscle. Compared with HUFA, feeding the HSFA diet up-regulated expression of *ACC*, *FAS*, *PPARγ,* and *SCD1*, but down-regulated expression of *CPT1B*. Compared with feeding HSFA, the HUFA diet led to greater concentrations of *c9*-C18:1 and other monounsaturated FA in LT muscle. Feeding HUFA also led to lower plasma concentrations of cholesterol, but there were no interactions between FA and lysophospholipids detected. Feeding lysophospholipids improved growth and feed conversion ratio and altered meat quality by increasing muscle pH_24h_, redness values (24 h), IMF content, and concentrations of C18:3, C20:5 and total polyunsaturated fatty acids. Furthermore, lysophospholipids supplementation led to lower malondialdehyde content and up-regulated the expression of *ACC*, *FAS,* and *LPL* in LT muscle.

**Conclusions:**

Results indicated that supplementing a high-concentrate diet with lysophospholipids to beef bulls can enhance growth rate, feed efficiency, meat quality, and beneficial FA. Increasing the dietary ratio of UFA to SFA reduced DM intake and backfat thickness without compromising growth, suggesting potential improvements in feed efficiency.

**Supplementary Information:**

The online version contains supplementary material available at 10.1186/s40104-024-01138-w.

## Background

According to a FAO report, the rapid expansion of the Chinese economy has resulted in a surge of beef consumption, effectively positioning China as the second-largest consumer of beef globally [1]. The projected 10% increase in per capita consumption by the Chinese population over the next decade represents a 50% rise relative to the previous decade. Despite this, domestic beef cattle production faces challenges due to the high breeding costs and lengthy growing periods, all of which have created a shortfall in output and an expanding gap between demand and supply [2]. Breaching the gap between demand and supply requires a heightened focus, not only on enhancing production efficiency amidst rising demand and production costs, but also on meeting the public demands for healthier beef with superior nutritional profiles and sensory attributes [3]. Consequently, optimizing feed efficiency and growth rates while also considering the impact of dietary intake on carcass and meat quality during the fattening period is a key priority for the beef cattle industry.

The quality characteristics of meat and meat products, including flavor, physicochemical properties, and shelf life, are closely linked to the quantity and composition of lipid in the meat [4]. Fatty acids (FA) play a critical role in determining the sensory properties of meat, affecting its tenderness, juiciness, and flavor [5]. Previous studies have explored the effects of diets supplemented with various rumen-protected lipid sources containing diverse FA profiles or varying the dietary ratio of unsaturated FA to saturated FA on meat quality and FA composition of beef [6, 7]. Despite this, there are still opportunities to conduct research with rumen-protected lipid sources enriched with specific FA and their effects on beef quality and FA profiles.

Palmitic acid (C16:0), stearic acid (C18:0), and oleic acid (*cis*-9 C18:1) are the predominant FA in both milk fat and adipose tissue, and constitute the main FA of commercial lipid supplements frequently fed to dairy cows [8]. For instance, feeding an *cis*-9 C18:1-rich diet enhanced FA digestion, absorption, and energy allocation in lactating cows, whereas dietary inclusion of C16:0 tended to enhance milk fat yield and energy output [9]. Individual FA also can exert differential effects on muscle FA composition and lipid metabolism. For instance, dietary enrichment with C18:1 increased oleic acid concentration in muscle tissue [10], whereas supplementation with C16:0 and C18:0 led to upregulation of lipogenesis-related genes such as *ACC* and *SCD1*, thereby enhancing adipose tissue deposition [11].

Lysophospholipids, a type of phospholipid, are typically found as single-chain acyl phospholipid derivatives through the hydrolysis or enzymatic breakdown of phospholipids [12]. In recent years, lysophospholipids have emerged as a promising feed additive with the potential to enhance production and feed efficiency in ruminants. Although research on muscle FA composition remains limited, it has been suggested that lysophospholipids exert regulatory effects on FA composition and muscle lipid concentration [13]. Huo et al. [14] reported that lysophospholipids supplementation to lambs decreased serum lipase levels, stimulated lipid deposition in muscle, and enhanced meat quality. The mechanism of activation whereby lysophospholipids modulate lipid metabolism and accumulation across various tissues involves the upregulation of genes associated with FA delivery, synthesis, and uptake [15].

Based on the limited aforementioned findings, it is evident that dietary supplementation with either FA or lysophospholipids holds promise for enhancing growth performance and meat quality of ruminants through the modulation of lipid metabolism. Furthermore, considering both FA and lysophospholipids have beneficial impacts on growth and lipid metabolism, the potential interactions between FA and lysophospholipids supplementation are worthy of study. In light of these considerations, we hypothesized that dietary supplementation of FA varying with ratios of UFA to SFA and lysophospholipids could improve growth performance, meat quality, and promote lipid deposition in muscle. Hence, the objective of this study was to investigate the effects of dietary supplementation with FA and lysophospholipids on growth performance, meat quality, plasma biochemical indices, FA profiles, oxidative stability, and lipid metabolism in finishing beef cattle.

## Materials and methods

### Animals, experimental design, and diets

Thirty-two Angus bulls [initial body weight (BW) = 623 ± 22.6 kg and aged 21 ± 0.5 months] were used in a finishing trial. The experiment was a completely randomized block design with a 2 × 2 factorial arrangement of treatments: 2 FA diets [High SFA diet (HSFA) vs. High UFA diet (HUFA)] combined with 2 levels of lysophospholipids supplementation [0 vs. 0.075%, dry matter (DM) basis]. Based on our previous study with minor modifications, the HSFA and HUFA diets were formulated to contain the same total lipid content but differed in UFA to SFA ratio, 1:2 and 1:1, respectively (Table S1) [3]. The bulls were blocked by BW and randomly allocated into 8 blocks of 4 bulls each. Within each block, bulls were randomly assigned to 1 of the 4 treatments. The supplemented dietary lipid was supplied by Yihai Kerry Food Industry Co., Ltd. (Tianjin, China) and it was in rumen-protected form as calcium salts of FA produced through the saponification reaction between FA and calcium oxide. The lysophospholipids product was supplied by Kemin Technology Co., Ltd. (Zhuhai, China) and contained phospholipids, free fatty acids, and lysophospholipids comprising 30% of the total. The lysophospholipids product consisted of single-chain acyl phospholipid derivatives produced through the hydrolysis of soybean lecithin. The supplemental dose of lysophospholipids was determined based on both the manufacturer's recommendations and our previous study [16]. The diets were formulated to meet the nutrient requirements of finishing beef bulls targeting a daily gain of 1.5 kg/d as recommended by NASEM [17]. The composition and nutrient profile of the experimental diets are detailed in Table 1. The bulls were housed individually in tie-stalls and fed a total mixed ration (TMR) at libitum twice daily at 08:00 and 17:00. The ration was prepared daily using a feed mixer (Data Ranger, American Calan Inc., Northwood, NH, USA). The experiment lasted 104 d, consisting of 14 d for adaptation and 90 d for data and sample collection.

**Table 1:** Ingredients and nutrient composition of dietary treatments

Item	HSFA^1^		HUFA	
	L−	L+	L−	L +
Ingredient composition, % of DM				
Corn grain	45.00	45.00	45.00	45.00
Soybean meal	8.50	8.50	8.50	8.50
Peanut hull	15.00	15.00	15.00	15.00
Corn stalk	15.00	15.00	15.00	15.00
Distillers dried grains with solubles	5.00	5.00	5.00	5.00
Corn germ meal	5.50	5.50	5.50	5.50
Salt (sodium chloride)	0.80	0.80	0.80	0.80
Limestone	1.00	1.00	1.00	1.00
Sodium bicarbonate	1.10	1.10	1.10	1.10
Magnesium oxide	0.20	0.20	0.20	0.20
Mineral-vitamin premix^2^	0.40	0.40	0.40	0.40
L^3^	0	0.075	0	0.075
Calcium salts of FA 1^4^	2.50	2.50	0	0
Calcium salts of FA 2^5^	0	0	2.50	2.50
Chemical composition, % of DM ^6^				
Dry matter, %	88.92	89.33	89.85	89.86
Organic matter	92.09	91.53	92.43	91.42
Crude protein	11.54	11.58	11.49	11.52
Ether extract	6.86	6.88	6.83	6.89
Neutral detergent fiber	30.80	31.16	30.69	30.92
Acid detergent fiber	18.53	18.75	18.22	18.90
Calcium	0.78	0.78	0.77	0.79
Phosphorus	0.38	0.38	0.38	0.39
NEm, Mcal/kg	1.76	1.79	1.74	1.76
NEg, Mcal/kg	1.14	1.16	1.12	1.13

^1^HSFA, UFA:SFA ratio of 1:2; HUFA, UFA:SFA ratio of 1:1; L−, diet without lysophospholipids supplementation; L+, diet supplemented with lysophospholipids at 0.075% (DM basis)

^2^The Mineral-vitamin premix provided the following per kilogram of the diet: vitamin A 6,000 IU, vitamin D 600 IU, vitamin E 50 IU, Fe 10 mg, Cu 15.0 mg, Mn 27 mg, Zn 65 mg, Se 0.10 mg, I 0.50 mg, Co 0.20 mg

^3^*L,* Lysophospholipids

^4^Including 62% C16:0, 15% C18:0, 15% C18:1, 3% C18:2

^5^Including 48% C16:0, 5% C18:0, 36% C18:1, 9% C18:2

^6^NEm and NEg levels were estimated according to NASEM [17]

### Sample collection

Daily feed intake was monitored by weighing the amount of feed offered and the refusals that were recorded daily before the morning feeding. Samples of feed and refusals were collected weekly, oven-dried at 55 °C for 48 h, and ground through a 1-mm screen using a standard model 4 Wiley Mill (Arthur H. Thomas, Philadelphia, PA, USA) for chemical analysis. Animals were weighed individually before the morning feeding on d 0, 45, and 90 and the average daily gain (ADG) was calculated based on the increased BW and the number of days on feed. Feed conversion ratio (FCR) was determined as the ratio of day matter intake (DMI) to ADG. Blood samples were collected from the tail vein of each bull into vacutainer tubes containing sodium heparin as an anticoagulant 4 h before the morning feeding on d 45 and 90. These blood samples were then centrifuged at 3,000 × *g* for 20 min at 4 °C to obtain plasma and were stored at −20 °C until analysis.

At the end of the experiment, the bulls were trucked to a local commercial abattoir (Changhao, Harbin, China), within 1 h distance, then slaughtered the following day. The animals were kept off feed but were given free access to water before slaughtering. All bulls were electrically stunned, exsanguinated, skinned, eviscerated, and split down the midline according to standard commercial procedures. The hot carcass weight (HCW) was recorded post-slaughter, and dressing percentage was calculated as HCW divided by final BW × 100. The backfat thickness was measured on the left side of the carcass between the 12^th^ and 13^th^ ribs, using a graduated caliper. The measurement extended three-fourths of the ribeye’s length from the cranial portion. After the carcass was chilled at 4 °C for 24 h, ribeye area (REA) was determined using a ruled grid as outlined by Silva et al. [18]. After determining the REA, samples of the *Longissimus thoracis* (LT) muscle were immediately collected between the 12^th^ and 13^th^ ribs and subdivided into 2 portions. One portion was stored at −20 °C for chemical composition and FA profile analysis. The remaining portion was stored at −80 °C for antioxidant and gene expression analyses.

### Laboratory analysis

#### Feed analysis

The composite diets, feed ingredients, and meat samples were analyzed for DM (method 930.15), organic matter (OM, method 942.05), and crude protein (CP, method 990.03) according to the Association of Official Analytical Chemists [19]. The neutral detergent fiber (NDF) and acid detergent fiber (ADF) content of feed samples were analyzed according to Van Soest et al. [20] with heat-stable amylase and sodium sulfite used in the NDF procedure. The contents of ether extract (method 920.39) in the diets and meat samples were determined according to AOAC [21].

#### Blood indices

The plasma concentrations of triglycerides (TG), cholesterol (CHOL), high-density lipoprotein cholesterol (HDL-C), and low-density lipoprotein cholesterol (LDL-C) were determined with a fully automatic biochemical analyzer using standard commercial kits (Nanjing Jiancheng Bioengineering Institute, Nanjing, China). The non-esterified FA (NEFA) were determined via an enzymatic method using commercial kits (Nanjing Jiancheng Bioengineering Institute, Nanjing, China) following manufacturer’s instruction.

#### Meat quality evaluation

The pH of the LT muscle was measured at 45 min and 24 h after slaughtering (with the sample stored in air at 4 °C for 24 h) by inserting a portable pH meter (HI9125; Hanna Instruments, Padova, Italy) with temperature compensation directly into the muscle. Meat color parameters, including redness (a*), yellowness (b*), and lightness (L*), were measured using a portable chromameter (CR-300, Minolta, Osaka, Japan). Cooking loss was evaluated following the method outlined by He et al. [22]. Briefly, the muscle was sliced into cubes measuring 6 cm × 4 cm × 4 cm, aligned parallel to the muscle fiber direction. Subsequently, the sample was weighed (*W*_1_), wrapped in polyethylene bags, and then heated in a water bath at 80 °C until the internal temperature reached 70 °C. After cooling to approximately 25 °C, the cooked sample was wiped with filter paper and reweighed (*W*_2_). The cooking loss (%) was calculated as:

$$\mathrm{Cooking}\;\mathrm{loss}\;\left(\%\right)\;=\;\left(W_{1}\;-\;W_{2}\right)\!/ W_{1} \times 100$$

Shear force was measured using a C-LM3-type Digital Muscle-Shear Apparatus (Harbin, China) following the protocol outlined by Silva et al. [18]. Drip loss was determined according to the method described by Meng et al. [15]. In brief, the meat samples were sliced into cubes measuring 3 cm × 3 cm × 3 cm and weighed as *W*_1_. These samples were then suspended and stored at 4 °C for 24 h, then blotted dry on filter paper, and reweighed as *W*_2_. Drip loss was expressed as the percentage of the difference between the initial and final weight divided by initial weight:

$$\mathrm{Drip}\;\mathrm{loss}\;\left(\%\right)\;=\;\left(W_{1}\;-\;W_{2}\right)\!/W_{1}\;\times\;100$$

#### Muscle antioxidants

Approximately 0.2 g of LT muscle sample was combined with 1.8 mL of physiological saline and homogenized using high-speed cryogenic grinding (BSH-C2, Hangzhou Suizhen Biotechnology Co., Ltd., Zhejiang, China) at 4 °C. The homogenate was then centrifuged at 3,500 r/min for 10 min to isolate the supernatant. The protein content of the supernatant was quantified using a BCA Protein Assay kit (Beyotime Biotechnology Institute, Shanghai, China) following the manufacturer's instructions. Additionally, total antioxidant capacity (T-AOC), the activities of catalase (CAT), superoxide dismutase (SOD), and glutathione peroxidase (GSH-Px), and the content of malondialdehyde (MDA) in LT muscle samples were analyzed using assay kits in accordance with the manufacturer's instructions (Nanjing Jiancheng Bioengineering Institute, Nanjing, China).

#### Fatty acid profiles

Dietary FA profiles were analyzed according to the method of Sukhija and Palmquist [23]. Feed samples were methylated with 5% methanol HCl, 6% potassium carbonate and hexane. Lipids were extracted from freeze-dried muscle samples using a chloroform–methanol mixture (2:1, v/v) according to the procedure described by Folch et al. [24]. The samples were transesterified into FAME as per the method detailed by He et al. [25]. The FAME analysis was conducted using an HP6890 gas chromatography system equipped with a SP-2560 capillary column (100 m × 0.25 mm × 0.20 µm). The oven temperature program followed these steps: from 150 to 160 °C at a rate of 1 °C/min, then increased to 167 °C at 0.2 °C /min, and finally raised to 225 °C at 1.5 °C/min, maintained at 225 °C for 5 min. The injector and detector temperatures were set at 250 °C. Hydrogen was used as the carrier gas at a flow rate of 1 mL/min, and 1 μL of sample was injected. Peaks in chromatograms were identified by comparison to reference standards from Supelco. The conjugated linoleic acid isomers and *trans*- and *cis*-octadecenoic acids were identified with reference to previous reports [26]. The FAME were quantified using an internal standard, nonadecanoic acid (C19:0) methyl ester, which was added to each sample prior to methylation.

#### Real-time-PCR (RT-PCR)

Total RNA was extracted from frozen tissues using Trizol reagent (Takara, Dalian, China) following the manufacturer's instructions. The purity and concentration of the total RNA were assessed using a spectrophotometer to ensure that the OD_260_/OD_280_ value fell within the range of 1.9 to 2.1. Subsequently, RNA was reverse-transcribed into cDNA using a reverse transcription kit (BL699A, biosharp, China) according to the manufacturer's protocols. Gene expression levels in each sample were determined using β-actin as an internal reference gene. The relative expression of each candidate gene was calculated using the 2^−ΔΔCt^ method, with all data normalized to the reference gene. The primer sequences used for synthesis are listed in Table 2.

**Table 2:** Primers used for quantitative real-time PCR

Genes	Primer sequence (5′→3′)	Product size, bp	Genbank No.
*ACC*	F-AGGAGGGAAGGGAA TCAGAA	69	NM_174224
	R- GCTTGAACCTGTCGGAAGAG		
*FAS*	F- A TCGAGTGCA TCAGGCAAGT	92	NM_001012669
	R- TGTGAGCACA TCTCGAAAGCC		
*CPT1B*	F-GCGACTCCAGTGGGACATTC	144	NM_001034349
	R-AAAGGCAGGAACTGGAAGCA		
*HSL*	F- GA TGAGAGGGTAA TTGCCG	100	NM_001080220
	R- GGA TGGCAGGTGTGAACT		
*PPARγ*	F- GTGAAGCCCA TTGAGGACAT	148	NM_181024
	R- AGCTGCACGTGTTCTGTCAC		
*LPL*	F- GGAGTGACCGAATCTGTGGCTAAC	181	NM_001075120
	R- GGCACCCAACTCTCATACATTCCTG		
*ATGL*	F-TCTGCCTGCTGATTGCTATG	121	FJ798978
	R-GGCCTGGATAAGCTCCTCTT		
*CD36*	F-GGTCCTTACACA TACAGAGTTCG	115	NM_174010
	R-A TAGCGAGGGTTCAAAGA TGG		
*SCD1*	F-TTA TTCCGTTA TGCCCTTGG	83	NM_173959
	R-TTGTCA TAAGGGCGGTA TCC		
*β-actin*	F-AGCAAGCAGGAGTACGATGAGT	120	NM_173979
	R-ATCCAACCGACTGCTGTCA		

*ACC* Acetyl-CoA carboxylase α, *FAS* Fatty acid synthase, *CPT1B* Carnitine palmitoyl-transferase 1B, *HSL* Hormone-sensitive lipase, *PPARγ* Peroxisome proliferators activated receptor γ, *LPL* Lipoprotein lipase, *ATGL* Adipose triglyceride lipase, *CD36* Fatty acid translocase, *SCD1* Stearoyl-CoA desaturase 1

### Statistical analysis

Model validation was performed using diagnostic plots, including fitted residuals and Q-Q plots, with R software. Where it was required, Box-Cox transformation was applied to enhance homogeneity and normality, and models were re-fitted using the transformed data. All data were subjected to analysis using the MIXED procedure of SAS 9.4 (SAS Institute Inc., Cary, NC, USA). The model encompassed fixed effects, including the FA diets (HSFA vs. HUFA), lysophospholipids supplementation, and the interaction between the FA diets and lysophospholipids with block as the random effect. The significance among treatments was evaluated using Tukey’s multiple comparison test when the interaction between diet FA and lysophospholipids was significant. Statistical significance was declared at *P* ≤ 0.05. Trends were considered at 0.05 < *P* ≤ 0.10 unless otherwise stated.

## Results

### Growth performance and carcass traits

No interaction between FA diet and lysophospholipids was observed for growth performance or carcass traits (Table 3). Feeding with the HUFA diet led to lower (*P* = 0.005) DMI compared with feeding HSFA, whereas the final BW, ADG and FCR were not affected by the FA diet. Supplementation of lysophospholipids did not affect the final BW and DMI but led to greater (*P* = 0.002) ADG and improved (*P* = 0.002) the FCR. Except for backfat thickness which was lower (*P* = 0.032) with HUFA compared with HSFA, carcass traits were not affected by FA diet or lysophospholipids supplementation.

**Table 3:** Effects of dietary SFA to UFA ratio and lysophospholipids supplementation on growth performance and carcass traits in beef bulls (*n* = 8)

Item^1^	HSFA^2^		HUFA		SEM	*P*-value		
	L−	L+	L−	L+		FA	L	FA × L
Growth performance								
Initial BW, kg	620	625	625	622	22.6	0.974	0.952	0.869
Final BW, kg	736	760	736	753	21.7	0.879	0.348	0.867
ADG, kg/d	1.28	1.50	1.24	1.45	0.06	0.466	0.002	0.987
DMI, kg/d	13.29	13.24	12.16	12.29	0.39	0.005	0.908	0.792
FCR	10.39	8.90	9.84	8.61	0.38	0.292	0.002	0.734
Carcass traits								
Hot carcass weight, kg	433	449	430	446	11.3	0.796	0.171	0.997
Dressing, %	58.92	59.15	58.45	59.19	0.84	0.802	0.570	0.764
Backfat thickness, cm	1.65	1.66	1.56	1.62	0.03	0.032	0.213	0.402
REA, cm^2^	103	102	104	103	3.9	0.744	0.907	0.746

^1^*DMI* Dry matter intake, *ADG* Average daily gain, *FCR* Feed conversion ratio, *REA* Ribeye area

^2^HSFA, UFA:SFA ratio of 1:2; HUFA, UFA:SFA ratio of 1:1; L−, Diet without lysophospholipids supplementation; L+, Diet supplemented with lysophospholipids at 0.075% (DM basis)

### Meat quality

There was no interaction between diet FA and lysophospholipids for meat quality (Table 4). Compared with the HSFA diet, feeding the HUFA diet to bulls led to greater (*P* < 0.001) shear force but it resulted in lower IMF content (*P* = 0.001). However, altering ratio of UFA to SFA did not change muscle pH, meat color, drip loss, cooking loss, or DM and CP content of LT muscle. The supplementation of lysophospholipids led to greater pH_24h_ (*P* = 0.007) and a* values (*P* = 0.029) whereas it resulted in lower L* values (*P* = 0.035) after 24 h post-slaughter and also cooking loss (*P* = 0.004). Although the contents of DM and CP in the LT muscle were not affected, the IMF content was greater (*P* = 0.027) with dietary lysophospholipids supplementation compared with the control (no lysophospholipids addition).

**Table 4:** Effects of dietary SFA to UFA ratio and lysophospholipids supplementation on meat quality in beef bulls (*n* = 8)

Item	HSFA^1^		HUFA		SEM	*P*-value		
	L−	L+	L−	L+		FA	L	FA × L
Muscle pH								
pH_45min_	6.67	6.69	6.61	6.72	0.08	0.846	0.607	0.473
pH_24h_	5.57	5.73	5.46	5.68	0.06	0.207	0.007	0.687
Color parameters								
L* (lightness) 45 min	29.37	28.02	30.35	28.79	0.87	0.330	0.113	0.905
a* (redness) 45 min	13.79	14.21	13.96	13.67	0.88	0.833	0.940	0.689
b* (yellowness) 45 min	3.94	4.29	4.12	3.72	0.55	0.728	0.960	0.498
L* (lightness) 24 h	35.88	33.82	35.35	33.60	0.84	0.659	0.035	0.856
a* (redness) 24 h	18.99	20.92	19.24	20.46	0.67	0.873	0.029	0.606
b* (yellowness) 24 h	10.91	11.13	10.98	11.62	1.57	0.896	0.789	0.857
Shear force, N	40.52	39.88	48.69	45.93	1.52	< 0.001	0.276	0.492
Drip loss, %	3.17	3.05	3.31	2.14	0.34	0.272	0.072	0.137
Cooking loss, %	33.78	31.76	33.61	29.53	0.92	0.205	0.004	0.278
Composition of the LT muscle								
Dry matter, %	31.40	31.85	32.21	33.23	1.51	0.179	0.512	0.388
Crude protein, % DM	22.23	21.80	21.98	22.49	1.38	0.238	0.466	0.925
Intramuscular fat, % DM	7.95	10.50	6.73	8.79	0.62	0.001	0.027	0.689

^1^HSFA, UFA:SFA ratio of 1:2; HUFA, UFA:SFA ratio of 1:1; L−, Diet without lysophospholipids supplementation; L+, Diet supplemented with lysophospholipids at 0.075% (DM basis)

### Plasma biochemical indices

Interactions between diet FA and lysophospholipids were observed for the blood concentration of NEFA (*P* = 0.011) (Table 5). Compared with the HSFA diet, feeding the HUFA diet led to lower concentrations of CHOL (*P* = 0.019) and LDL-C (*P* = 0.001). Blood concentration of NEFA was not affected by lysophospholipids supplementation in the HSFA diet, whereas it was lower (*P* < 0.05) when supplementing lysophospholipids with the HUFA diet. Furthermore, the supplementation of lysophospholipids also led to lower (*P* = 0.006) plasma TG concentration.

**Table 5:** Effects of dietary SFA to UFA ratio and lysophospholipids supplementation on plasma lipid indices in beef bulls (*n* = 8)

Item^1^	HSFA^2^		HUFA		SEM	*P*-value		
	L−	L+	L−	L+		FA	L	FA × L
TG, mmol/L	0.22	0.20	0.22	0.18	0.10	0.423	0.006	0.167
CHOL, mmol/L	4.18^a^	3.76^b^	3.64^b^	3.67^b^	0.12	0.019	0.133	0.087
HDL-C, mmol/L	2.92	2.83	2.72	2.74	0.11	0.170	0.704	0.583
LDL-C, mmol/L	0.58	0.54	0.47	0.47	0.02	0.001	0.430	0.358
NEFA, mmol/L	0.13^b^	0.13^b^	0.18^a^	0.14^b^	0.01	0.004	0.045	0.011

^1^*TG* Triglyceride, *CHOL* Cholesterol, *HDL-C* High-density lipoprotein cholesterol, *LDL-C* Low-density lipoprotein cholesterol, *NEFA* Non-esterified FA

^2^HSFA, UFA:SFA ratio of 1:2; HUFA, UFA:SFA ratio of 1:1; L−, Diet without lysophospholipids supplementation; L+, Diet supplemented with lysophospholipids at 0.075% (DM basis)

### Antioxidant status

There was no interaction between diet FA and lysophospholipids addition for antioxidants in LT muscle (Table 6). Compared with the HSFA diet, the content of SOD was lower (*P* = 0.016) and the MDA concentration was greater (Trend; *P* = 0.073) by feeding the HUFA diet. Furthermore, the dietary lysophospholipids supplementation led to lower (*P* = 0.002) muscle MDA content and tended to increase (*P* = 0.078) T-AOC. The contents of CAT and GSH-Px in LT muscle were not affected by diet FA or lysophospholipids addition.

**Table 6:** Effects of dietary SFA to UFA ratio and lysophospholipids supplementation on antioxidative status in *Longissimus thoracis* from beef bulls (*n* = 8)

Item^1^	HSFA^2^		HUFA		SEM	*P*-value		
	L−	L +	L−	L+		FA	L	FA × L
T-AOC, U/mg protein	0.45	0.46	0.45	0.48	0.01	0.441	0.078	0.659
SOD, U/mg protein	48.45	46.64	42.88	44.86	1.397	0.016	0.952	0.190
CAT, U/mg protein	4.92	5.30	5.17	4.95	0.30	0.881	0.787	0.334
GSH-Px, U/mg protein	114	111	114	109	7.4	0.945	0.564	0.894
MDA, nmol/mg protein	1.98	1.69	2.24	1.80	0.10	0.073	0.002	0.465

^1^*T-AOC* Total antioxidative capacity, *SOD* Superoxide dismutase, *CAT* Catalase, *GSH-Px* Glutathione peroxidase, *MDA* Malonaldehyde

^2^HSFA, UFA:SFA ratio of 1:2; HUFA, UFA:SFA ratio of 1:1; L−, Diet without lysophospholipids supplementation; L+, Diet supplemented with lysophospholipids at 0.075% (DM basis)

### Fatty acid composition

Interactions between diet FA and lysophospholipids supplementation were not noticed for FA profiles in LT muscle (Table 7). Greater ratio of UFA to SFA in bull diets led to lower content of C16:0 (*P* = 0.001) and greater content of *c9*-C18:1 (*P* = 0.004) without changing the other FA profiles, as a result, the sum of SFA was lower (*P* = 0.009) and MUFA was greater (*P* = 0.021) in the LT muscle. In addition, supplementing lysophospholipids in diets led to greater contents of C18:3 (*P* = 0.001), C20:5 (*P* = 0.001) and consequently increased the contents of n-3 PUFA (*P* = 0.002) and total PUFA (*P* = 0.050), but decreased (*P* = 0.003) the n-6/n-3 PUFA ratio.

**Table 7:** Effects of dietary SFA to UFA ratio and lysophospholipids supplementation on fatty acid profiles in *Longissimus thoracis* from beef bulls (g/100 g total fatty acids) (*n* = 8)

Item^1^	HSFA^2^		HUFA		SEM	*P*-value		
	L−	L+	L−	L+		FA	L	FA × L
C14:0	2.15	2.02	2.03	2.01	0.15	0.664	0.608	0.712
C15:0	0.25	0.25	0.24	0.24	0.01	0.629	0.915	0.913
C16:0	29.33	29.09	27.99	28.01	0.36	0.001	0.727	0.667
*c9*-C16:1	3.33	3.39	3.08	3.22	0.19	0.466	0.721	0.880
C17:0	0.69	0.68	0.68	0.67	0.014	0.421	0.643	0.884
*c9*-C17:1	0.57	0.57	0.5	0.55	0.04	0.727	0.847	0.885
C18:0	17.68	17.30	16.77	16.68	0.72	0.295	0.746	0.847
*c9*-C18:1	39.91	40.15	42.50	42.03	0.67	0.004	0.861	0.610
*c11*-C18:1	0.83	0.85	0.83	0.81	0.03	0.384	0.844	0.416
*c9,c12* C18:2, n-6	2.12	2.15	2.18	2.15	0.14	0.862	0.994	0.830
*c9,t11*-CLA^2^	0.22	0.21	0.19	0.21	0.01	0.238	0.659	0.329
*t10,c12-* CLA^2^	0.13	0.12	0.12	0.10	0.01	0.522	0.483	0.955
C18:3, n-3	0.80	1.68	0.82	1.11	0.07	0.754	0.001	0.532
*c9*-C20:1	0.12	0.11	0.10	0.11	0.02	0.119	0.716	0.257
C20:3, n-6	0.17	0.17	0.17	0.18	0.01	0.455	0.732	0.945
C20:4, n-6	0.59	0.59	0.59	0.62	0.03	0.542	0.690	0.647
C20:5, n-3	0.22	0.29	0.22	0.31	0.02	0.541	0.001	0.749
C22:5, n-3	0.96	0.91	0.91	1.04	0.109	0.721	0.729	0.440
C22:6, n-3	0.11	0.12	0.11	0.12	0.020	0.832	0.545	0.898
SFA	50.10	49.34	47.71	47.61	0.82	0.009	0.548	0.644
MUFA	44.77	45.08	47.08	46.73	1.17	0.021	0.977	0.676
PUFA	5.19	5.61	5.20	5.71	0.24	0.818	0.050	0.841
n-3 PUFA	2.07	2.49	2.06	2.58	0.13	0.776	0.002	0.704
PUFA/SFA	0.10	0.11	0.11	0.12	0.01	0.274	0.060	0.945
n-6/n-3 PUFA	1.53	1.53	1.53	1.23	0.08	0.892	0.003	0.889

^1^*CLA* Conjugated linoleic acid, *SFA* Saturated fatty acids, *MUFA* Monounsaturated fatty acids, *PUFA* Polyunsaturated fatty acids

^2^HSFA, UFA:SFA ratio of 1:2; HUFA, UFA:SFA ratio of 1:1; L−, Diet without lysophospholipids supplementation; L+, Diet supplemented with lysophospholipids at 0.075% (DM basis)

### Lipid-metabolic genes expression

**Fig. 1:**
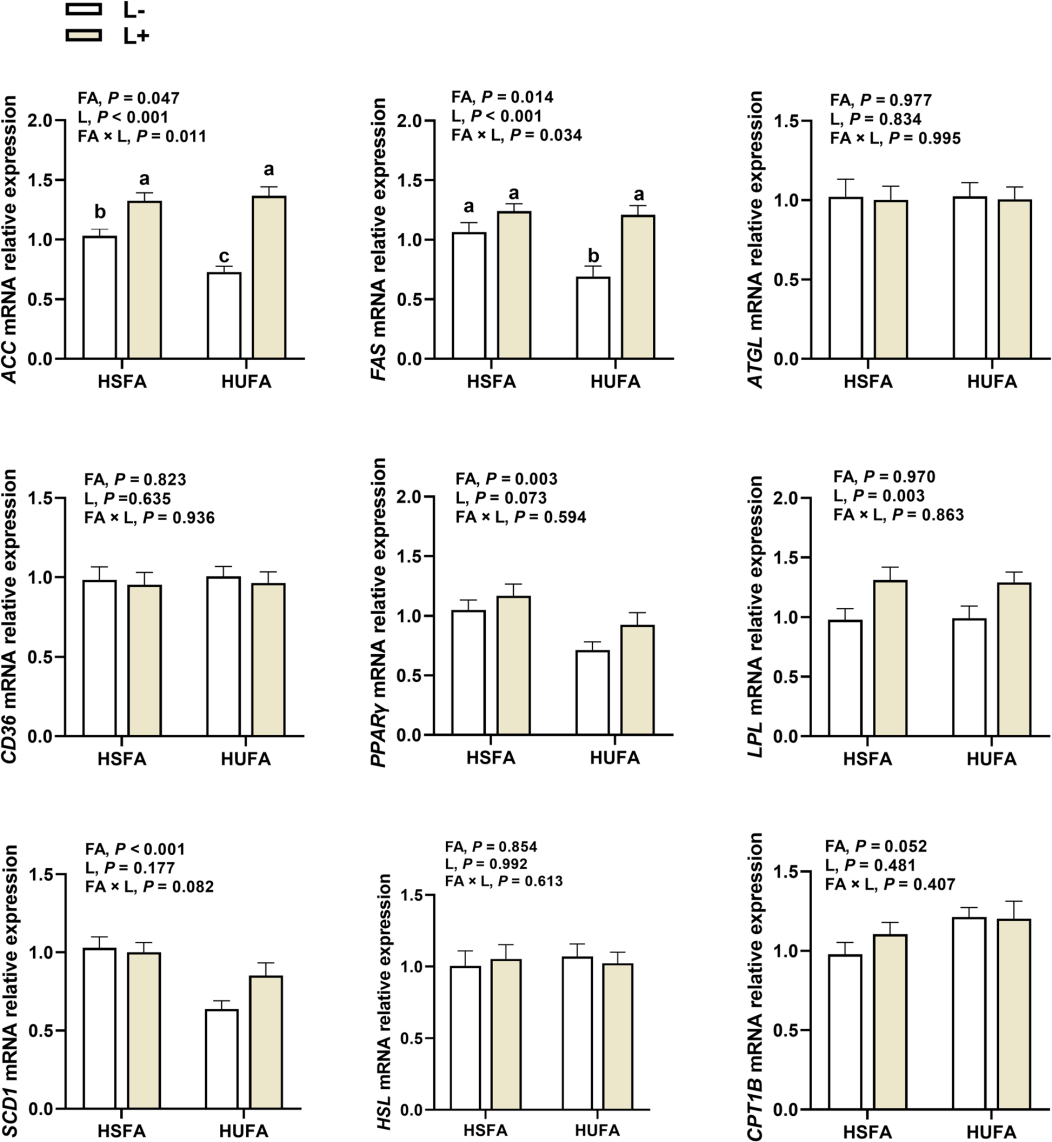
Effects of dietary SFA to UFA ratio and lysophospholipids supplementation on lipid-metabolic genes expression in *Longissimus thoracis* from beef bulls. *ACC* Acetyl-CoA carboxylase α, *FAS* Fatty acid synthase, *ATGL* Adipose triglyceride lipase, *CD36* Fatty acid translocase, *PPARγ* Peroxisome proliferators activated receptor γ, *LPL* Lipoprotein lipase, *SCD1* Stearoyl-CoA desaturase 1, *HSL* Hormone-sensitive lipase, *CPT1B* Carnitine palmitoyl-transferase 1B. HSFA, UFA:SFA ratio of 1:2; HUFA, UFA:SFA ratio of 1:1; L−, Diet without lysophospholipids supplementation; L+, Diet supplemented with lysophospholipids at 0.075% (DM basis). The mRNA expressions were normalized to *β-actin* gene expression. All values are expressed as mean ± SEM (n = 8)

Interactions between diet FA and lysophospholipids were not observed except for *ACC* (*P* = 0.011) and *FAS* (*P* = 0.034) (Fig. 1). The expression of *ACC* and *FAS* was down-regulated (*P* < 0.05) by increasing ratio of UFA to SFA in the diet in the absence of lysophospholipids supplementation, whereas they did not differ between the HSFA and HUFA diets when lysophospholipids was added. In addition, compared with feeding HSFA, feeding the HUFA diet down-regulated the expression of *PPARγ* (*P* = 0.003) and *SCD1* (*P* < 0.001), while it led to a tendency for greater (*P* = 0.052) expression of *CPT1B*. Furthermore, the expression of *PPARγ* (Trend; *P* = 0.073) and *LPL* (*P* = 0.003) were up-regulated by supplementing lysophospholipids.

## Discussion

### Growth performance and carcass traits

Supplementation of individual FA such as palmitic (C16:0), stearic (C18:0), oleic (*cis*-9 C18:1), and linoleic acids (*cis*-9, *cis*-12 C18:2) is commonly done in dairy cow diets to enhance energy density and milk production [9]. However, some studies have highlighted that the inclusion of these FA can yield inconsistent results [27, 28]. Bai et al. [3] demonstrated that feeding diets with high concentrations of C16:0 and C18:0 promoted growth performance and meat quality of Angus bulls, suggesting that FA saturation may play a significant role in determining the growth performance and meat quality of beef cattle. In the current study, feeding HUFA diet to beef bulls resulted in lower DMI compared with the HSFA diet, which is consistent with findings from a previous study [9] that assessed the effect of several specific FA on nutrient digestibility, energy partitioning, and production responses of dairy cows. The impact of lipid supplements on DMI varies and is typically associated with the source of lipid. The suppressive effect of UFA on appetite in dairy cows is more pronounced compared with SFA, with DMI linear decreases reported as the degree of unsaturation of FA increases [8]. The reduced DMI of beef bulls due to higher UFA content in diets in the present study might be partially explained by potentially increased secretion of gut peptides associated with satiety such as cholecystokinin and glucagon-like peptide-1 [29]. However, despite a lack of change in the FCR, the lower DMI without reduced final BW and ADG suggests a potentially greater nutrient digestibility or an improvement of energy use efficiency by feeding HUFA versus HSFA as observed previously [8]. In fact, the favorable propensity of C18:1 for energy storage in the body was reported in dairy cows [9]. The lower backfat thickness with the HUFA diet indicated a reduction in energy cost for growth. The reduction in backfat thickness may be linked to the preferential mitochondrial transport and β-oxidation of UFA rather than SFA [30].

The present results of greater ADG and lower FCR in finishing beef bulls by supplementing dietary lysophospholipids is in agreement with previous studies that have highlighted the potential of lysophospholipids addition to enhance weight gain and feed efficiency [31, 32]. Reis et al. [32] demonstrated that incorporating lysophospholipids into milk replacers had favorable effects on the growth performance of dairy calves. Chen et al. [33] reported that supplementation of soybean lecithin rich with UFA led to greater ADG and lower FCR in steers. In the present study, the greater growth performance due to lysophospholipids supplementation could be attributed to improve nutrient digestibility. The lysophospholipids was suggested to facilitate micelle formation and enhance nutrient digestibility, thereby improving growth performance [12]. Furthermore, our previous investigation revealed that lysophospholipids supplementation led to greater concentration of butyrate in the digestive tract of beef steers, potentially promoting weight gain [12].

### Meat quality

The bulls fed the HUFA diet had greater shear force, a response that was consistent with the decreased IMF content. Gajaweera et al. [34] reported that IMF content is generally positively correlated with tenderness and negatively correlated with Warner–Bratzler shear force in Hanwoo beef cattle. The IMF plays a pivotal role in the palatability of beef by directly influencing tenderness, juiciness, and flavor [35]. In the current study, the lower IMF content with HUFA than HSFA diet may be attributed to the differences in FA metabolism within the muscle. Additionally, the reduction of backfat thickness with the HUFA diet is consistent with the decrease in IMF content, as both exhibit similar genetic correlations [36]. The SFA, particularly palmitic acid (16:0) and stearic acid (18:0), strongly stimulate adipogenic gene expression in intramuscular preadipocytes, whereas the monounsaturated FA (MUFA) oleic acid (*cis*-9 18:1) suppresses adipogenic gene expression [11]. Consistent with these findings, the expression of the adipogenic genes *ACC*, *FAS*, *PPARγ*, and *SCD1* was up-regulated in bulls fed the HSFA diet compared with those fed HUFA in the current study.

Beef quality is commonly assessed based on meat color, pH value, cooking loss, and sensory attributes (tenderness, juiciness, and flavour-likeness). Muscle pH is a critical indicator of meat quality because it influences shear force, water-holding capacity, and meat color [37]. In the present study, the greater muscle pH with addition of lysophospholipids indicated a reduction of muscle glycolysis rate because the meat pH is closely linked to the rate of muscle glycolysis [38]. Research suggests that lower concentrations of short-chain fatty acids (SCFAs) in feces may disrupt normal bacterial ecology and promote the phosphorylation of AMPK in myotubes and skeletal muscle, thus potentially resulting in greater rates of glycolysis [39, 40]. In fact, our previous study demonstrated that adding lysophospholipids in beef steer diets led to greater fecal total SCFA concentration [12] that would potentially decrease glycolysis rate and delay the post-mortem pH decline, thus, explaining the higher pH_24h_ with lysophospholipids supplementation in the present study.

Meat color plays a significant role in consumer purchasing decisions, with buyers in Western industrialized countries typically favoring a bright cherry-red color in beef. Brownish or other off-colors are commonly perceived as discoloration, which can lead to product rejection by consumers [41]. Thus, enhancing meat color is essential for improving meat quality and encouraging consumer purchasing of meat products. In the current study, lysophospholipids supplementation led to greater a* value while decreasing the L* value of beef meat. Similarly, several studies reported higher a* value and lower L* value in pigs fed lecithin containing a source of lysophospholipids [15, 42]. An association of high meat pH with a decrease of cooking losses was previously reported [43], and was confirmed in the present study by the greater pH and lower cooking losses with supplementation of lysophospholipids. Tenderness is a crucial palatability characteristic of meat, typically evaluated by shear force. It is known that IMF accumulation can induce intramuscular connective tissue remodeling, reducing collagen cross-linking and contributing to meat tenderization [44]. However, our results did not reveal a clear linkage between IMF content and shear force in bulls receiving diets supplemented with lysophospholipids. Nevertheless, the greater IMF content in the LT muscle with dietary supplementation of lysophospholipids aligns with the results of Li et al. [45] who reported greater fat content in the *Longissimus dorsi* of steers fed a diet containing lysophospholipids.

### Plasma biochemical indices

Elevated blood concentrations of TG, CHOL, and LDL-C may contribute to excessive fat accumulation in the liver, resulting in hepatic lipidosis, particularly during the fattening period when beef cattle have high energy demands [10]. In the current study, the decreased plasma TG, CHOL, and LDL-C concentrations by feeding the HUFA compared with the HSFA diet suggested that higher supplementation of C18:1 may effectively mitigate the risks associated with a high SFA diet in beef cattle. Our finding is supported by previous research demonstrating that MUFA such as C18:1 can reduce plasma concentrations of TG, CHOL, and LDL-C [46]. Harvatine and Allen [47] reported that Holstein cows fed diets supplemented with UFA had greater plasma NEFA concentrations compared with diets supplemented with SFA, which is consistent with our results of greater blood NEFA concentration by increasing dietary UFA concentration without the addition of lysophospholipids. This greater NEFA concentration with UFA supplementation may be attributed to enhanced UFA absorption, resulting in reduced insulin concentrations and enhanced lipolysis in adipose tissue [48]. Notably, the observed interactions between diet FA and lysophospholipids supplementation for CHOL (Trend) and NEFA concentrations is of interest; the lysophospholipids supplementation led to lower CHOL concentration when feeding HSFA, and to lower NEFA concentration when feeding HUFA, suggesting that the effect of lysophospholipids on plasma lipid metabolism may be influenced by the dietary FA composition. Consistently, He et al. [49] also reported that the addition of 0.05% lysophospholipids led to lower plasma CHOL concentrations in dairy cows. The mechanism by which lysophospholipids affects plasma biochemical parameters that change with the dietary ratio of UFA to SFA remains unclear, and further investigation is needed.

### Antioxidative status

Lipid oxidation is an important factor in the deterioration of meat quality, negatively affecting its flavor, color, and nutritional value, thus, impacting the overall quality of meat products [50]. Ruminants in the fattening phase are often fed high-concentrate diets, which can lead to subacute ruminal acidosis. This condition can disrupt free radical metabolism and cause an excessive production of reactive oxygen species (ROS). The accumulation of ROS in muscle tissue can impair the activity of metabolic enzymes and lead to significant oxidative damage to muscle cells [51]. Mitigating lipid oxidation and enhancing antioxidant enzyme activity are effective strategies for bolstering meat quality and extending its shelf life. Notably, in the current study, bulls fed the HUFA diet exhibited lower SOD activity compared with those fed HSFA. This disparity could stem from the greater unsaturation of the HUFA diet potentially compromising oxidative stability and increasing susceptibility to oxidation [52]. As a by-product of lipid peroxidation, MDA serves as a key biomarker for evaluating oxidative damage [12]. The trend of greater MDA in LT with HUFA than HSFA indicated a greater susceptibility to oxidative damage. Zhang et al. [53] reported that increasing the supplemental dose of lysophospholipids resulted in a linear decrease in the blood MDA concentration of beef cattle. The present study corroborates this finding, demonstrating that dietary lysophospholipids supplementation led to lower MDA content and a trend toward greater T-AOC activity. This outcome suggests that lysophospholipids could enhance antioxidant capacity thereby improving meat quality and extending shelf life, in particularly when higher UFA diets are fed because the magnitude of reduction in MDA by lysophospholipids (−18%) was more than the increase of MDA when feeding the UFA diet. In alignment with our results, Meng et al. [15] have elucidated lecithin's antioxidant properties and its synergistic effects with other antioxidants in preventing tissue oxidative damage. The observed enhancement in antioxidant capacity due to dietary lysophospholipids supplementation can be attributed in part to choline, a primary constituent of lysophospholipids, which regulates cellular redox states and suppresses inflammatory reactions [54].

### Fatty acid composition

The fatty acid profile is a key component that determines nutritional value and flavor of beef, with the SFA considered as major contributors to heart and vascular diseases for human health, while PUFA offer cardioprotective benefits [45]. Consequently, more consumers are not only focusing on the taste of meat but are also seeking healthier meat with high PUFA or MUFA content. In the current study, the higher proportion of *c9*-C18:1 and lower proportion of C16:0 in LT muscle of bulls fed the HUFA diet than the HSFA diet were expected because of the greater ratio of UFA to SFA in the HUFA vs. HSFA diets. Our results confirm that dietary manipulation of rumen bypass fat represents an effective nutritional approach for regulating the FA composition in beef. Our findings align with the study of Bai et al. [55] who observed that increasing dietary C18:1 content elevated the C18:1 content in the *longissimus dors*i muscle of finishing bulls. Additionally, the greater PUFA content, particularly increasing the contents of C18:3n-3 (α-Linolenic acid) and C20:5n-3 (EPA) in the LT muscle due to lysophospholipids supplementation are especially interest. In fact, α-Linolenic acid acts as a precursor for the synthesis of EPA and C22:6 n-3 (DHA), and both EPA and DHA have various health benefits. For example, the EPA and DHA play important regulatory roles for improving cardiovascular function and regulating inflammation in the human body [56, 57]. A previous study reported that the greater content of PUFA in muscle may be attributed to the protective effects of antioxidants in the diet [58]. Thus, the greater PUFA content in LT muscle with lysophospholipids supplementation may bolster the antioxidant activity of muscle thereby providing protection against PUFA peroxidation. Similarly, in line with our results, Li et al. [45] illustrated that feeding dietary soy lecithin to beef steers led to greater content of 20:5 n-3 in the *longissimus dorsi* muscle.

### Lipid metabolism gene expression

The present study revealed an increase in *PPARγ* expression in the LT muscle when bulls were fed the high SFA (HSFA diet) versus the low SFA (HUFA diets). As a transcription factor within the nuclear receptor superfamily, *PPARγ* plays a pivotal role in adipogenesis by facilitating adipocyte differentiation and fat deposition [59]. The present result suggests that SFA may enhance cell differentiation in bulls. These findings are in agreement with the observation by Bionaz et al. [60] that *PPARγ* is primarily activated by SFA, with UFA exhibiting only partial or null activation and even suppression. Additionally, it is known that *FAS* and *ACC* play pivotal roles as key enzymes in de novo FA synthesis [58]. In the current study, the expression of *ACC* and *FAS* was significantly down-regulated in bulls fed the HUFA vs. HSFA diet. Previous research has indicated that exogenous addition of C18:1 inhibited *ACC* expression in adipose tissue [61], implying that C18:1 may not be conducive to de novo FA synthesis. Interestingly, there was no difference in *ACC* and *FAS* expression between HSFA and HUFA diets supplemented with lysophospholipids, suggesting that lysophospholipids might mitigate the inhibition of *ACC* expression when the proportion of C18:1 increases. The enzyme encoded by *SCD1* is responsible for desaturating de novo synthesized or directly ingested C18:0 to MUFA [62]. A study reported that C16:0 and C18:0 promote *SCD1* expression in intramuscular preadipocytes, while C18:1 exerts a negative effect on its expression [11]. These findings are consistent with the present finding that *SCD1* expression in LT muscle of bulls fed HSFA diet was up-regulated. These results suggest that SFA may be more beneficial to endogenous FA synthesis than UFA. The HSL and CPT1B are rate-limiting enzymes in FA catabolism [54]. Inhibition of *SCD* catalytic activity has been shown to reduce the expression of genes involved in de novo FA synthesis while increasing the expression of genes involved in FA oxidation such as *CPT1B* [63]. This partly explains the decrease in *SCD1* expression and the increase in *CPT1B* expression observed in bulls fed HUFA diet in the current study.

Lipoprotein lipase serves as a rate-limiting enzyme, catalyzing the hydrolysis of triacylglycerol, thus, providing FA and monoacylglycerol for tissue storage or utilization [64]. In the current study, supplementation with lysophospholipids resulted in greater expression of *LPL*, *ACC*, and *FAS* suggesting a potential role for lysophospholipids in promoting muscle fat deposition. The upregulation of lipoprotein lipase, *ACC*, and* FAS* expression may be associated with the greater IMF content with lysophospholipids addition. Previous studies have reported a positive correlation between the expression of *ACC*, *FAS*, lipoprotein lipase, and IMF content [54, 65]. Our findings of increased expression of *FAS* by supplementing lysophospholipids are consistent with the results of Huang et al. [65] who demonstrated that dietary supplementation of lecithin led to a dose-dependent increase in* FAS* mRNA expression.

## Conclusion

Supplementing the diet of bulls with greater levels of SFA increased the IMF content and enhanced the antioxidant capacity in the LT muscle. Furthermore, elevated dietary SFA content also up-regulated the expression of lipid synthesis genes while down-regulated the expression of lipolytic genes in the LT muscle. Conversely, diets rich in UFA led to greater concentrations of C18:1 and MUFA in the LT muscle, thereby improving the FA composition of LT muscle. More importantly, the supplementation of high-concentrate diet with lysophospholipids revealed beneficial impacts on ADG, feed efficiency, meat quality and muscular antioxidant capacity, and promotion of fat deposition by up-regulating the expression of liposynthesis gene. Lastly, the overall absence of the interaction between diet FA and lysophospholipids supplementation indicated that both FA and lysophospholipids play roles in an independent manner. These results indicate that manipulating dietary UFA and SFA content have limited impact on growth and carcass traits, but could effectively alter beef quality and FA profiles. The lysophospholipids has evident potential for use in high-concentrate feeding of beef cattle for improving production efficiency and meat quality. Further studies focussed on more comprehensive measurements are warranted.

## Supplementary Information

Additional file 1. Table S1 Calculated fatty acid composition of the experimental diet. 

## Data Availability

The datasets produced and/or analyzed during the current study are available from the corresponding author on reasonable request.

## Abbreviations

ACC: Acetyl-CoA carboxylase α
ADF: Acid detergent fiber
ADG: Average daily gain
ATGL: Adipose triglyceride lipase
BW: Body weight
CAT: Catalase
CD36: Fatty acid translocase
CHOL: Cholesterol
CLA: Conjugated linoleic acid
CP: Crude protein
CPT1B: Carnitine palmitoyl-transferase 1B
DM: Dry matter
DMI: DM intake
FA: Fatty acids
FAS: Fatty acid synthase
FCR: Feed conversion ratio
HCW: Hot carcass weight
HDL-C: High-density lipoprotein cholesterol
HSFA: High SFA diet
HSL: Hormone-sensitive lipase
HUFA: High UFA diet
IMF: Intramuscular fat
GSH-Px: Glutathione peroxidase
LDL-C: Low-density lipoprotein cholesterol
LPL: Lipoprotein lipase
LT: *Longissimus thoracis*
MDA: Malondialdehyde
MUFA: Monounsaturated fatty acids
NDF: Neutral detergent fiber
NEFA: Non-esterified FA
OM: Organic matter
PPARγ: Peroxisome proliferators activated receptor γ
PUFA: Polyunsaturated fatty acids
REA: Ribeye area
ROS: Reactive oxygen species
SCFA: Short-chain fatty acid
SFA: Saturated FA
SOD: Superoxide dismutase
SCD1: Stearoyl-CoA desaturase 1
T-AOC: Total antioxidant capacity
TG: Triglycerides
TMR: Total mixed ration
UFA: Unsaturated FA
