# Supplementary material for: Impact of dietary lysophospholipids supplementation on growth performance, meat quality, and lipid metabolism in finishing bulls fed diets varying in fatty acid saturation
Source: J Anim Sci Biotechnol. 2025 Jan 9;16:7. doi: 10.1186/s40104-024-01138-w (PMC11715738; doi:10.1186/s40104-024-01138-w)
Supplement: Supplementary file 1 — Additional file 1. Table S1 Calculated fatty acid composition of the experimental diet. [file 40104_2024_1138_MOESM1_ESM.docx]

**Impact of** **dietary** **lysophospholipids supplementation on growth performance, meat quality, and** **lipid metabolism** **in finishing bulls fed diets varying in fatty acid saturation**

Meimei Zhang^1^, Haixin Bai^1^, Ruixue Wang^1^, Yufan Zhao^1^, Wenzhu Yang^2^, Jincheng Liu^1^, Yonggen Zhang^1⁎^, Peixin Jiao^1⁎^

^1^*College of Animal Science and Technology, Northeast Agricultural University, Harbin, 150030,* *People’s Republic of China*

^2^*Lethbridge Research and Development Centre, Lethbridge, T1J 4B1, Canada*

Correspondence: zhangyonggen@sina.com (Yonggen Zhang); peixin.jiao@neau.edu.cn (Peixin Jiao)

**Table S1** Calculated fatty acid composition of the experimental diet^1^

| **Fatty acids, %** | **HSFA** | |  | **HUFA** | |
| --- | --- | --- | --- | --- | --- |
|  | **L-** | **L+** |  | **L-** | **L+** |
| C10:0 | 0 | 0 |  | 0.15 | 0.20 |
| C12:0 | 0.13 | 0.15 |  | 1.61 | 1.89 |
| C14:0 | 0.85 | 0.87 |  | 1.33 | 1.36 |
| C16:0 | 50.82 | 50.51 |  | 37.31 | 36.20 |
| C17:0 | 0.16 | 0.16 |  | 0.38 | 0.12 |
| C18:0 | 13.90 | 14.12 |  | 6.72 | 6.29 |
| C18:1 | 22.61 | 22.40 |  | 39.10 | 39.1 |
| C18:2 | 9.70 | 9.81 |  | 12.00 | 11.2 |
| C20:1 | 0.28 | 0.27 |  | 0.28 | 0.34 |
| C20:2 | 0.29 | 0.27 |  | 0.36 | 0.55 |
| C21:0 | 0.88 | 0.89 |  | 1.01 | 2.49 |
| C22:1 | 0.32 | 0.36 |  | 0.28 | 0.32 |
| Others | 0.16 | 0.13 |  | 0.29 | 0.53 |
| UFA | 33.2 | 33.11 |  | 52.02 | 51.51 |
| MUFA^2^ | 23.21 | 23.03 |  | 39.66 | 39.76 |
| PUFA^3^ | 9.99 | 10.08 |  | 12.36 | 11.75 |
| SFA | 66.90 | 66.83 |  | 48.80 | 49.08 |
| UFA: SFA ratio | 1:2 | 1:2 |  | 1:1 | 1:1 |

^1^HSFA, UFA:SFA ratio of 1:2; HUFA, UFA:SFA ratio of 1:1; L-, Diet without lysophospholipids supplementation; L+, Diet supplemented with lysophospholipids at 0.075% (DM basis)

^2^*MUFA* Monounsaturated fatty acid

^3^*PUFA* Polyunsaturated fatty acid
